# β-Glucans and Resistant Starch Alter the Fermentation of Recalcitrant Fibers in Growing Pigs

**DOI:** 10.1371/journal.pone.0167624

**Published:** 2016-12-02

**Authors:** Sonja de Vries, Walter J. J. Gerrits, Mirjam A. Kabel, Thava Vasanthan, Ruurd T. Zijlstra

**Affiliations:** 1 Animal Nutrition Group, Wageningen University, Wageningen, The Netherlands; 2 Laboratory of Food Chemistry, Wageningen University, Wageningen, The Netherlands; 3 Department of Agricultural Food, and Nutritional Science, University of Alberta, Edmonton, AB, Canada; Colorado State University, UNITED STATES

## Abstract

Interactions among dietary ingredients are often assumed non-existent when evaluating the nutritive value and health effects of dietary fiber. Specific fibers can distinctly affect digestive processes; therefore, digestibility and fermentability of the complete diet may depend on fiber types present. This study aimed to evaluate the effects of readily fermentable fibers (β-glucans and resistant starch) on the degradation of feed ingredients containing more persistent, recalcitrant, fibers. Six semi-synthetic diets with recalcitrant fibers from rapeseed meal (pectic polysaccharides, xyloglucans, and cellulose) or corn distillers dried grain with solubles (DDGS; (glucurono)arabinoxylans and cellulose) with or without inclusion of β-glucans (6%) or retrograded tapioca (40%) substituted for corn starch were formulated. Six ileal-cannulated pigs (BW 28±1.4 kg) were assigned to the diets according to a 6×6 Latin square. β-glucan-extract increased apparent total tract digestibility (ATTD) of non-glucosyl polysaccharides (accounting for ~40% of the fiber-fraction) from rapeseed meal (6%-units, *P*<0.001), but did not affect non-glucosyl polysaccharides from DDGS. Retrograded tapioca reduced ATTD of non-glucosyl polysaccharides from rapeseed meal and DDGS (>10%-units, *P*<0.001), indicating that the large amount of resistant starch entering the hindgut was preferentially degraded over recalcitrant fibers from rapeseed meal and DDGS, possibly related to reduced hindgut-retention time following the increased intestinal bulk. Fermentation of fiber sources was not only dependent on fiber characteristics, but also on the presence of other fibers in the diet. Hence, interactions in the gastrointestinal tract among fibrous feed ingredients should be considered when evaluating their nutritive value.

## Introduction

Health claims and recommendations for fiber intake are based on the physiological effects of fibers that may indirectly affect energy intake and utilization, gut health, and risks for certain diseases [1–3]. The generic term “dietary fiber”, however, covers a variety of structures with manifold chemical and physical properties [4]. Hence, the nutritive value and potential beneficial health effects of fibers will depend on their specific characteristics. When evaluating the nutritive value and health effects of dietary fiber, effects of different fiber types that are present in food are usually assumed to be additive [2]. Also, in animal nutrition, the nutritive value of fibers from feed ingredients is predicted based on the total quantity of fibers in each ingredient and interactions among feed ingredients are assumed to be absent [5]. Although this approach is convenient and allows making general recommendations for fiber intake, independent of diet composition, the validity of this assumption should be questioned. Specific fiber types can have distinct effects on digestive processes, such as through their modulating effects on physicochemical properties and gastrointestinal transit of digesta, and on microbial colonization in the gastrointestinal tract [6]. For example, fermentable fibers may increase digesta mass entering the hindgut, reducing digesta retention time in the large intestine, potentially decreasing the extent of fermentation of the dietary fiber fraction [7–9]. Viscous fibers such as soluble β-glucans and arabinoxylans, can increase (gastric) retention time–although effects differ among fiber sources and levels [10, 11]–, prolonging the time available for nutrient degradation in the stomach. In contrast, viscous fermentable fibers may also reduce apparent nutrient digestibility in the small intestine, due to reduced enzyme accessibility, interactions with the gut mucosa, or increased microbial activity [12–14]. Hence, digestibility and fermentability of the complete diet can be affected by the presence of specific fibers, where the direction of effects will vary among fiber types. It is important to evaluate the effects of specific fibers and account for the potential effects of other fiber sources or other dietary components.

The objective of the present study was to test the hypothesis that the presence of readily fermentable fibers in the diet alters the degradation of fibers that are more resistant to degradation in the porcine gastro-intestinal tract (i.e. recalcitrant fibers). Rapeseed meal (RSM) and distillers dried grain with solubles (DDGS) were chosen as model feedstuffs, containing fibers rich in pectic polysaccharides, xyloglucans, and cellulose (RSM) or (glucurono-)arabinoxylans and cellulose (DDGS). Both fiber fractions are rather recalcitrant and only partly fermented by the porcine microbiota (30–70% of non-starch polysaccharides) (30–70% of non-starch polysaccharides; [15, 16, 17]), but the microbial populations involved in their degradation may differ. β-Glucans and resistant starch were selected for their high extent of fermentation and different ability to modulate physicochemical digesta properties, to study their effect on the fermentability of fibers from RSM and DDGS. Viscous β-glucans can increase gastric retention time of soluble digesta and are potentially fermented in the small intestine [11, 18, 19]. Hence, β-glucans were hypothesized to enhance fermentation of RSM fibers and to a lesser extent that of DDGS fibers, because of its stimulation of microbial activity. In contrast, resistant starch was expected to reduce fermentation of RSM and DDGS fibers, due to reduced digesta retention time following the increased ileal and large intestinal bulk. A novel approach, separating glucosyl-containing non-starch polysaccharides (NSP) from non-glucosyl polysaccharides [20] was used to allow particular evaluation of the effects of the added β-glucans and resistant starch on the fermentation of RSM and DDGS fibers. As both β-glucans and resistant starch exclusively consist of glucosyl-residues, degradation of these added fiber sources could be separated from degradation of non-glucosyl polysaccharides originating from RSM or DDGS.

## Materials and Methods

### Materials and diets

Effects of the presence of β-glucans and resistant starch in diets on nutrient and fiber degradability of RSM (*Brassica napus*) and DDGS were tested in a 2 × 3 factorial arrangement. Two basal diets, containing either 500 g/kg RSM or DDGS and ~400 g/kg corn starch were formulated to meet or exceed nutrient requirements of growing pigs [5]. Corn starch was partly replaced with the β-glucan-extract (β-GLUC; 60 g/kg w/w) or completely replaced with retrograded tapioca (RG; 400 g/kg w/w), resulting in 6 dietary treatments (Table 1). The β-GLUC (Agri-Food Discovery Place, University of Alberta, Edmonton, AB, Canada) was extracted from barley (*Hordeum vulgare L*.) flour (CDC Rattan hulless barley, Tomtene Seed Farm, Birch Hill, SK, Canada); the resistant starch source consisted of retrograded starch (RS3; C*ActistarTM 11700, Cargill, Haubourdin, France) obtained by crystallizing hydrolysed tapioca (maltodextrins), originating from cassava (*Manihot esculenta*) root [21].

**Table 1:** Composition and physicochemical properties of experimental diets containing either 500 g/kg rapeseed meal or corn distillers dried grain with solubles (DDGS), with or without inclusion of β-glucan-extract (β-GLUC) or retrograded tapioca (RG)*,^†^.

	Rapeseed meal			DDGS		
Item	Control	β-GLUC	RG	Control	β-GLUC	RG
Ingredient, g/kg as-fed						
Rapeseed meal^‡^	500	500	500	-	-	-
Corn DDGS^§^	-	-	-	500	500	500
Corn starch^‖^	413	235	-	433.5	255.5	-
β-GLUC^¶^	-	200	-	-	200	-
RG^**^	-	-	413	-	-	433.5
Caseinate^††^	22	-	22	22	-	22
Rapeseed oil	30	30	30	-	-	-
Sodium bicarbonate	7	7	7	7	7	7
Potassium bicarbonate	-	-	-	5	5	5
Limestone	5	5	5	5	5	5
Dicalcium phosphate	5	5	5	5	5	5
Salt	4	4	4	4	4	4
Mineral and vitamin premix^‡‡^	10	10	10	10	10	10
L-Lysine HCL	2	2	2	4	4	4
DL-Methionine	-	-	-	0.7	0.7	0.7
L-Threonine	-	-	-	1	1	1
L-Tryptophan	-	-	-	0.8	0.8	0.8
TiO_2_	2	2	2	2	2	2
Analyzed chemical composition, g/kg DM^§§^						
DM, g/kg as-fed	912	914	920	925	923	929
Crude protein	230	212	213	177	173	165
Crude fat	52	55	54	53	54	49
Total glucosyl	528	518	553	579	594	608
Free glucose	n.d.^‖‖^	2	n.d.^‖‖^	3	6	6
Starch	413	309	446	454	341	471
β-glucans	1	58	1	3	63	4
Residual glucosyl polysaccharides^¶¶^	61	97	50	61	126	68
Non-glucosyl polysaccharides	71	100	67	105	116	99
Rhamnosyl	1	2	2	1	1	0
Arabinosyl	23	33	21	31	36	30
Xylosyl	9	24	9	44	49	41
Mannosyl	4	6	3	9	12	9
Galactosyl	8	9	8	8	8	8
Uronyl	25	27	24	10	10	10
Water binding capacity, g/g DM	2.32	3.58	2.38	1.85	2.52	1.90
*In vitro* viscosity, mPa·s	1.28	60.0	1.37	1.63	11.8	1.62

DDGS, distillers dried grain with solubles; β-GLUC, β-glucan-extract; RG, retrograded tapioca; n.d., not detected.

^*^ Corn starch (control diet) was either replaced with β-GLUC (~200 g/kg, as-fed basis) or RG (~400 g/kg, as-fed basis).

^†^ Rapeseed meal diets provided per kilogram of diet: 18.7 MJ GE, 15.2 MJ DE, 2.5, 10.6, 7.7, 6.7, and 2.2 g apparent ileal digestible phosphorus, lysine, methionine+cysteine, threonine, and tryptophan, respectively. Distillers dried grain with solubles diets provided per kilogram of diet: 18.5 MJ GE, 15.3 MJ DE, 3.5, 10.5, 7.1, 6.4, and 2.1 g apparent ileal digestible phosphorus, lysine, methionine+cysteine, threonine, and tryptophan, respectively. Calculated based on data from Centraal Veevoeder Bureau [5] and Widmer et al. [33].

^‡^ *Brassica napus* meal. For composition see S1 Table.

^§^ High protein corn DDGS. For composition see S1 Table.

^‖^ Native corn starch.

^¶^ β-glucan-extract from barley flour (CDC Rattan hull-less barley, Tomtene Seed Farm, Birch Hill, SK, Canada). Per 100 g. the extract contained 4 g moisture, 3 g ash, 6 g crude protein, 24 g starch, and 49 g NSP of which 27 g β-glucans. For more information on composition see S1 and S2 Tables.

^**^ Retrograded Tapioca (C*Actistar^TM^ 11700; Cargill, Haubourdin, France). For information on composition S1 and S2 Tables.

^††^ Emser 14 (DMV International, Veghel, The Netherlands).

^‡‡^ Provided per kilogram of diet: Cu, 50 mg, as CuSO_4_•H_2_O; Fe, 75 mg, as FeSO_4_·H_2_O; I, 0.5 mg, as KI; Mn, 25 mg, as MnO; Se, 0.3 mg, as Na_2_SeO_3_; Zn, 125 mg, as ZnSO_4_·H_2_O; Vitamin A (retinyl acetate), 2.5 mg; vitamin D (cholecalciferol), 19 μg; vitamin E (DL-α-tocopherol), 33 mg; vitamin B_1_ (thiamin), 2.5 mg; vitamin B_2_ (riboflavin), 5 mg; vitamin B_6_ (pyridoxine-HCl), 1.5 mg; vitamin K_3_ (menadione), 4 mg; vitamin B_12_ (cyanocobalamin), 15 μg; niacin, 37.5 mg; pantothenic acid (d-calcium pantothenate), 15 mg; biotin, 250 mg; folic acid, 2.5 mg;

^§§^ Unless indicated otherwise.

^‖‖^ Not detected, with detection limit at 0.2 g/100g as analyzed.

^¶¶^ Calculated as total glucosyl minus free glucose and glucosyl from starch and β-glucans.

### Animals and experimental procedures

The animal experiment was conducted at the Swine Research and Technology Centre at the University of Alberta (Edmonton, AB, Canada). The animal protocol was approved by the Animal Care Committee of the University of Alberta and followed the guidelines established by the Canadian Council on Animal Care [22]. At the end of the study, pigs were humanly euthanized by captive bold stunning, immediately followed by exsanguination. Four pigs lost their cannula during the experiment and were euthanized prior to the experimental endpoint.

A total of 10 crossbred barrows (initial body weight, 28 ± 1.4 kg (SD); Duroc × Large White/Landrace; Hypor, Inc., Regina, SK, Canada) were moved 1 week before surgery into individual metabolism pens (1.2 × 1.2 m). Each pen was equipped with a single-space feeder and a low-pressure bowl drinker. Pigs were surgically fitted with a T-cannula (inner diameter 2.0 cm) at the distal ileum. Ten days post-surgery, six pigs were randomly assigned the six experimental diets according to a 6 × 6 Latin square design. The four remaining pigs served as reserve and replaced experimental animals when required. In total, four pigs had to be replaced during the experiment due to loss of cannulas and the experimental period was extended with one period. Pigs were replaced so that over seven periods minimally two observations were realized for each pig and each diet was tested at least in six periods. Hence, 46 observations were obtained in 10 pigs over 7 periods in an incomplete 10 × 7 Youden square. Diets were fed as mash and mixed with water (1:3) in the feed through. The daily feed allowance was 2.6 × energy requirements for maintenance (460 kJ digestible energy (DE)/kg of BW^0.75^, based on the calculated DE content of the control RSM or DDGS diet) in 2 equal meals at 08.00 and 15.00 h. All pigs consumed their complete allowance during the entire experiment. Pigs had free access to water. After a 5 d gradual transition from starter to experimental diets, the experiment, consisting of 7 sequential periods, started. Each 14 d experimental period consisted of a 9 d adaption to the diets followed by 2 d collection of feces, 2 d collection of ileal digesta for digestibility measurements, and 1 d collection of ileal digesta for the measurement of retention time. Pigs were weighed weekly during the experiment after consuming their morning meal. Feces were collected from 08.00 to 17.00 h using bags attached to rings glued around the anus. Bags were collected within 1 h after defecation and immediately frozen (-20°C). Ileal digesta for digestibility measurements were collected from 08.00 to 17.00 h into plastic bags (10 cm in length and 4 cm in diameter). The bags were removed when filled approximately 70% with digesta, or after a maximum of 1 h, and immediately frozen (-20°C). For retention time measurements, on day 14 of each experimental collection, 3.4 g Cr as Cr_2_O_3_ (solid phase marker) and 3.4 g Co as Co (II)-EDTA (soluble phase marker) were mixed into the morning meal. Digesta were collected at 45, 90, 180, 270, 360, 540, and 720 min after feed consumption. At each time-point, cannulas were opened and digesta were collected into plastic bags during 5 min. If a sample was not obtained after 5 min, bags were left on until digesta were collected or until the next collection time point. The actual time of collection was recorded for each sample.

Digesta and feces were thawed, pooled per pig within period, subsampled, and immediately frozen (-20°C). Digesta and feces samples for digestibility measurements were freeze-dried.

### Analytical methods

Water-binding capacity (WBC) of diets was analyzed in duplicate by soaking 1 g of material in 25 ml of water for 24 h at room temperature. Samples were centrifuged (3274 *g* for 20 min) and decanted [23]. Water binding capacity was calculated as the weighed quantity of water retained per gram of dry material. Before viscosity analyses, diets were diluted with distilled water (1:2, w/w), incubated for 3 h at 37°C, centrifuged (12000 *g* for 10 min), and decanted. Digesta samples were thawed, centrifuged (12000 *g* for 10 min), and decanted. *In vitro* apparent viscosity of supernatants was analyzed before and after heating (80°C, 10 min) in duplicate using a rheometer (UDS 200; Paar Physica, Glenn, VA, USA) at a shear rate of 1.29/s, 12.9/s and 129/s and a temperature of 20°C [24].

Before chemical analyses, individual freeze-dried samples were ground in a centrifugal mill using a 0.5-mm screen (model ZM1, Retsch; Brinkman Instruments, Rexdale, ON, Canada). All chemical analyses were performed in duplicate using standard laboratory methods [25, 26]. Diets, digesta, and feces were analyzed for content of dry matter (DM; AOAC 930.15), titanium (AOAC 540.91), nitrogen (AOAC 990.03; using a Thermo Quest NA 2100 Nitrogen and Protein Analyzer, Interscience B.V., Breda, The Netherlands), diethyl ether extract after hydrochloric acid hydrolysis (AOAC 920.39; using Soxhlet apparatus and petroleum diethyl ether), free glucose and total starch (AOAC 996.11; using a commercial test kit, Megazyme International Ltd., Ireland), mixed linked β-glucans (using a commercial test kit, Megazyme International Ltd., Ireland), total glucosyl residues, and total complex carbohydrates.

Total complex carbohydrates were measured as neutral sugars and uronic acids after removal of small soluble saccharides [27]. Before analyses of neutral sugar and uronic acid contents in diets, NSP were extracted from diets [28]. Briefly, starch was gelatinized and enzymatically degraded, subsequently NSP were precipitated using acidified ethanol Neutral sugar composition was analyzed according to the method of Englyst and Cummings [29]. Briefly, after pre-treatment with 72% (w/w) H_2_SO_4_ for 1 h at 30°C, samples were hydrolysed with 1 M H_2_SO_4_ at 100°C for 3 h. Constituent monosaccharides were derivatized into their corresponding alditol acetates and analyzed using gas chromatography (Focus-GC, Thermo Scientific, Waltham, MA). Inositol was used as an internal standard. Uronic acid content was analyzed according to the automated colorimetric m-hydroxydiphenyl assay [30], using an auto-analyzer (Skalar Analytical B.V., Breda, The Netherlands). Galacturonic acid was used for calibration.

Residual glucosyl polysaccharides in diets were calculated as total glucosyl residues minus free glucose and glucosyl residues originating from starch and β-glucans. Crude protein content was calculated as N (%, w/w) × 6.25 (ISO 5983 [26]) unless indicated otherwise.

Digesta samples needed for retention time measurements were thawed, dried (70°C for 16 h, 103°C for 4 h; AOAC 930.15), incinerated (550°C for 3 h; AOAC 942.05), and analyzed for content chromium and cobalt after acid hydrolysis[31] using a SpectrAA 300 atomic absorption spectrophotometer (Varian B.V., Middelburg, The Netherlands).

### Calculation and statistical analysis

Apparent ileal digestibility (AID) and apparent total tract digestibility (ATTD) were calculated, using TiO_2_ as indigestible marker [32]. Mean retention time (MRT) of digesta until the terminal ileum was calculated using Cr_2_O_3_ (solid phase) or Co-EDTA (soluble phase) markers [9].

Data were analyzed using a generalized linear mixed model with beta-distributed error for the response variable and a logit link function (PROC GLIMMIX, SAS version 9.2, SAS Institute Inc., Cary, NC). A normal distributed error and identity link was assumed for AID of non-glucosyl polysaccharides, a gamma distributed error and log link for ileal viscosity and MRT. Pigs within period were the experimental units. Feed ingredient (RSM or DDGS), fiber source (β-GLUC or RG), their interaction, and period were included as fixed effects. Pig was included as random R-side effect to account for repeated observations within a pig. Significance of approximate F-tests for fixed effects was determined by the Residual Pseudo Likelihood method using Newton-Raphson Ridge Optimization; degrees of freedom were approximated using the Kenward-Roger method. Model assumptions and goodness of fit of the model and covariance structure were evaluated through the distribution of residuals and the ratio of the obtained generalized Chi-square to the degrees of freedom. Preliminary analyses identified best fit of a heterogeneous autoregressive covariance (ARH (1)) structure for AID and ATTD of residual glucosyl polysaccharides and non-glucosyl polysaccharides, MRT, and ileal viscosity, and a compound symmetry (CS) structure for all other parameters. Significance of differences was tested using type III pseudo-likelihood ratio statistics. Contrasts were used to compare β-GLUC- and RG-diets with control diets and to compare the average chromium to cobalt ratio in ileal digesta from the 6 diets to a baseline value. Data are presented as back-transformed means and pooled standard deviation unless stated otherwise. Differences among means with *P* < 0.05 were considered significant.

## Results

### Diets

Analyzed chemical composition of feed ingredients and diets are presented in Tables 1 and 2, S1 and S2 Tables. Water binding capacity and *in vitro* viscosity were greater in the β-GLUC diets than in the control and RG diets (Table 1).

**Table 2:** Apparent ileal and total tract digestibility of DM and nutrients in growing pigs fed diets containing either 500 g/kg rapeseed meal or corn distillers dried grain with solubles (DDGS), with or without inclusion of β-glucan-extract (β-GLUC) or retrograded tapioca (RG)*,^†^.

	Rapeseed meal			DDGS				*P*-value^‡^			
							Pooled		Fiber source		
	Control	β-GLUC	RG	Control	β-GLUC	RG	SD	I	β-GLUC	RG	I x F
n^§^	10 (7)	7 (7)	7 (6)	8 (7)	7 (6)	7 (6)					
Apparent ileal digestibility, %											
DM	69.5	59.6	46.5	71.4	61.8	46.3	1.90	0.014	<0.001	<0.001	0.14
Crude protein	74.7	71.2	75.1	72.8	68.0	70.3	2.22	<0.001	<0.001	0.09	0.06
Crude fat	82.1^b^	80.4^b^	84.7^a^	77.0^x^	71.5^y^	75.5^x^	2.76	<0.001	<0.001	0.34	0.04
Total glucosyl	93.1^a^	82.8^b^	50.3^c^	91.2^x^	82.3^y^	52.7^z^	2.25	0.23	<0.001	<0.001	0.03
Starch	99.6^a^	98.9^b^	54.7^c^	97.6^x^	95.8^y^	51.5^z^	2.09	<0.001	0.003	<0.001	<0.001
β-glucans	n.a.	23.7	n.a.	n.a.	24.9	n.a.	10.45				
Residual glucosyl polysaccharides^‖^	77.8^a^	82.5^a^	32.1^b^	77.5^x^	82.8^x^	53.5^y^	8.51	0.013	0.06	<0.001	0.004
Non-glucosyl polysaccharides^¶^	-26.5^b^	-8.7^a^	-33.5^b^	19.3^x^	5.0^x^	3.4^x^	14.53	<0.001	0.76	0.07	0.040
Arabinosyl	-0.5	7.7	-1.5	18.5	5.8	12.7	11.33	0.006	0.62	0.46	0.06
Xylosyl	-6.2^b^	14.9^a^	-26.7^c^	27.2^x^	7.9^z^	15.8^y^	5.59	0.001	0.037	0.002	0.002
Uronyl	-50.1^a^	-57.5^a^	-54.6^a^	-35.9^x^	-58.6^y^	-86.9^z^	22.32	0.127	0.027	<0.001	<0.001
Apparent total tract digestibility, %											
DM	83.4^a^	80.9^b^	80.5^b^	85.1^x^	81.9^y^	79.9^z^	1.22	0.038	<0.001	<0.001	0.021
Crude protein	82.6	76.4	73.8	82.0	73.0	68.4	2.94	<0.001	<0.001	0.001	0.11
Crude fat	78.3^a^	75.9^b^	77.0^ab^	72.6^x^	66.9^y^	66.6^y^	2.08	<0.001	<0.001	<0.001	0.031
Total glucosyl	98.0	97.6	97.1	96.8	96.8	96.0	0.68	<0.001	0.40	0.001	0.65
Starch	99.9^a^	99.8^b^	99.8^b^	99.9^x^	99.8^y^	99.6^z^	0.06	0.033	<0.001	<0.001	0.044
β-glucans	n.a.	99.9	n.a.	n.a.	99.9	n.a.	0.03				
Residual glucosyl polysaccharides^‖^	92.2	93.5	91.4	87.4	91.2	86.1	2.38	<0.001	0.03	0.29	0.50
Non-glucosyl polysaccharides^¶^	70.3	76.1	59.7	51.4	52.3	34.5	4.95	<0.001	0.040	<0.001	0.13
Arabinosyl	86.5	88.5	82.7	55.5	58.8	44.0	4.23	<0.001	0.09	0.004	0.65
Xylosyl	84.5	91.2	75.8	43.3	49.3	27.9	6.14	<0.001	0.004	<0.001	0.38
Uronyl	50.9	54.6	45.6	54.7	49.4	40.7	6.61	0.27	0.73	<0.001	0.10

DDGS, distillers dried grain with solubles; β-GLUC, β-glucan-extract; RG, retrograded tapioca; I, ingredient; F, fiber source; n.a., not analyzed.

^a,b ^When an interaction between feed ingredient and fiber source was detected, superscripts (^a,b,c^ for RSM or ^x,y,z^ for DDGS) indicate differences among diets within a feed ingredient (*P* < 0.05).

^*^ Corn starch (control diet) was either replaced with β-glucan-extract (β-GLUC; ~60 g/kg, as-fed basis) or retrograded tapioca (~400 g/kg, as-fed basis).

^†^ Data are back transformed least square means and pooled SD, except for digestibility values of β-glucan that are presented as raw means and pooled SD.

^‡^ Model established *P*-values for fixed effects of feed ingredient (I, RSM vs. DDGS), fiber source (β-GLUC or RG vs. control) and the interaction between feed ingredient and fiber source (I x F).

^§^ Number of replicate observations. Values between parentheses indicate the number of pigs in which replicate observations were made. In total, 46 observations were realized in 10 pigs over 7 periods.

^‖^ Calculated as total glucosyl minus glucosyl from starch and β-glucans.

^¶^ Monosaccharides represent anhydrous sugar moieties.

### Apparent ileal and total tract degradability

The AID and ATTD of crude protein, crude fat, and starch of the RSM diets were greater (*P* < 0.05) compared with the DDGS diets (Table 2). The AID of non-glucosyl polysaccharides of the RSM diets was negative and less (*P* < 0.01) than that of the DDGS diets, whereas ATTD was greater (*P* < 0.01). β-GLUC reduced AID and ATTD of DM, crude protein, and starch in the RSM and the DDGS diets. The AID and ATTD of crude fat were only reduced (*P* < 0.01) by β-GLUC in the DDGS diet. β-GLUC increased ATTD of non-glucosyl polysaccharides in both diets (*P* = 0.04), particularly so in the RSM diet. RG reduced (*P* < 0.10) AID and ATTD of DM, starch, and non-glucosyl polysaccharides in RSM and DDGS diets. The AID of crude protein was less in the RG diet compared with the control diet for DDGS (2% units) but not for RSM (I × F, *P* = 0.06). RG increased AID of crude fat in the RSM but not in the DDGS diet (I × F, *P* = 0.04). RG did not affect ATTD of crude fat in the RSM diet, whereas it reduced ATTD of crude fat in the DDGS diet (I × F, *P* = 0.03).

### Digesta kinetics

Mean retention time in the stomach and small intestine of the soluble phase was greater (7 to 68 min, *P* = 0.02) for DDGS diets compared with RSM diets whereas mean retention time of the solid phase did not differ (Table 3). β-GLUC and RG both reduced mean retention time of the solid phase in the RSM and the DDGS diets (23 to 57 min, *P <* 0.04). Viscosity of digesta in the ileum was less (*P* < 0.05) in DDGS control and RG diets compared with RSM diets (Table 3). β-GLUC increased viscosity measured in heated supernatant (*P <* 0.01), whereas RG did not affect viscosity.

**Table 3:** Mean retention time in the stomach and small intestine of solid and soluble digesta fractions and viscosity of ileal digesta in growing pigs fed diets containing either 500 g/kg rapeseed meal (RSM) or corn distillers dried grain with solubles (DDGS), with or without inclusion of β-glucan-extract (β-GLUC) or retrograded tapioca (RG)*,^†^.

	Rapeseed meal			DDGS				*P*-value^‡^			
							Pooled		Fiber source		
Item	Control	β-GLUC	RG	Control	β-GLUC	RG	SD	I	β-GLUC	RG	I x F
n^§^	10 (7)	7 (7)	7 (6)	8 (7)	7 (6)	7 (6)					
Mean retention time solid phase^ǁǁ^, min	388	336	336	372	315	349	34.0	0.28	0.002	0.020	0.22
Mean retention time soluble phase^¶^, min	268	284	288	293	335	295	43.1	0.021	0.13	0.50	0.40
Viscosity^**^, mPa·s	1.65	1.63	1.62	1.33	1.65	1.45	0.24	0.029	0.07	0.52	0.10
Viscosity (heated)^††^, mPa·s	3.24	21.50	3.53	2.73	235.7	2.90	40.8	<0.001	<0.001	0.48	<0.001

DDGS, distillers dried grain with solubles; β-GLUC, β-glucan-extract; RG, retrograded tapioca; I, ingredient; F, fiber source.

* Corn starch (control diet) was either replaced with β-glucan-extract (β-GLUC; ~60 g/kg, as-fed basis) or retrograded tapioca (~400 g/kg, as-fed basis).

† Data are back-transformed least square means and pooled SD.

^‡^ Model established *P*-values for fixed effects of ingredient (I, RSM vs. DDGS), fiber source (β-GLUC or RG vs. control) and the interaction between feed ingredient and fiber source (I x F).

^§^ Number of replicate observations. Values between parentheses indicate the number of pigs in which replicate observations were made. In total, 46 observations were realized in 10 pigs over 7 periods.

^ǁǁ^ Mean retention time in the stomach and small intestine measured using a solid phase marker (Cr_2_O_3_).

^¶^ Mean retention time in the stomach and small intestine measured using a soluble phase marker (Co-EDTA).

** Viscosity measured in digesta supernatant at 20°C, immediately following thawing, at shear rate of 129/s.

^††^ Viscosity measured in digesta supernatant after heating (80°C, 10 min) at shear rate of 129/s.

## Discussion

The results of the present study confirmed our hypothesis that addition of β-glucans or resistant starch to fiber-rich diets affects digesta properties, nutrient digestion, and gastrointestinal retention time of digesta, and interacts with the degradation of recalcitrant fiber fractions from RSM (pectic polysaccharides, xyloglucans, and cellulose) and DDGS ((glucurono)arabinoxylans and cellulose). The novel approach to separate glucosyl-containing NSP from non-glucosyl polysaccharides enabled the particular evaluation of the effects of added β-glucans and resistant starch on the non-glucosyl fiber fractions from DDGS and RSM. As both β-glucans and resistant starch exclusively consist of glucosyl residues, the degradation of these added glucan-fiber sources can be separated from the degradation of non-glucosyl polysaccharides originating from RSM or DDGS. This non-glucosyl polysaccharides fraction represents 60% (RSM) or 64% (DDGS) of the NSP in these feedstuffs, corresponding to ~40% (RSM) or ~60% (DDGS) of the total dietary fiber fraction. Due to the addition of β-GLUC, non-glucosyl polysaccharides represented a smaller amount of NSP in the β-GLUC diets (~50%). We calculated residual glucosyl polysaccharides, by subtracting glucosyl residues from starch and β-glucans from the total glucosyl fraction, representing cellulose and other glucosyl-containing NSP. In RSM-diets, non-cellulosic glucosyl-containing polysaccharides accounted for ~30% of residual glucosyl polysaccharides and originated mainly from xyloglucans [34, 35], whereas in DDGS the majority of residual glucosyl polysaccharides originated from cellulose and only minor amounts from substitutions to the arabinoxylans [36–38].

### Nutrient digestibility of RSM and DDGS diets

Apparent ileal digestibility of crude protein from RSM was calculated to be ~72% and AID of crude protein from DDGS ~68% (calculated by difference method; AID for caseinate assumed to be 96.2%, AID for synthetic amino acids assumed to be 100% [5]). As expected, crude protein from RSM was better digested than crude protein from DDGS [5]. The lower crude fat digestibility of DDGS compared with RSM diets was likely caused by the rapeseed oil included in RSM diets, whereas crude fat in the DDGS diets originated completely from the DDGS (Table 1). Similarly, the presence of, presumably partly resistant, starch in DDGS (4.5%, S1 Table) can explain the lower AID of starch in the DDGS diets compared with the RSM diets. The lower ATTD of crude fat compared with AID of crude fat, indicates net microbial synthesis of fat in the hindgut[39].

### Fiber degradability

Non-starch polysaccharides can be measured as the sum of constituent monosaccharides released by acid hydrolysis of samples after removal of small soluble saccharides by ethanol [29]. Due to the high starch content of the diets, their NSP content is generally measured in NSP extracts after starch removal [27]. Calculations based on analyzed NSP contents of individual feed ingredients, however, indicate that some soluble NSP may be removed along with the starch, resulting in slight underestimation of NSP contents measured in NSP extracts of diets (S2 Table). For the current diets, especially glucosyl-polysaccharides were underestimated (1 to 4%, w/w, S2 Table), whereas underestimation of non-glucosyl polysaccharides was less (0 to 2% w/w, data not shown).

**Fig 1:**
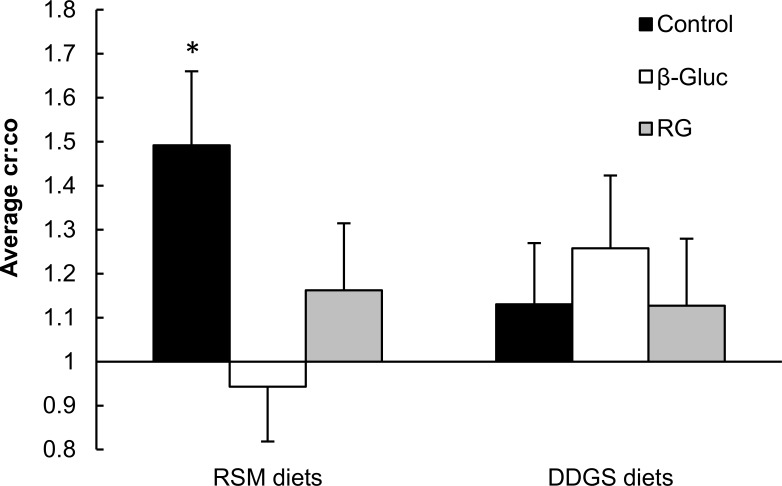
Average chromium to cobalt ratio in ileal digesta of pigs collected during 12 h after feeding a pulse dose of Cr_2_O_3_ and Co-EDTA markers. Digesta samples were collected at 45, 90, 180, 270, 360, 540, and 720 min after feed consumption. Baseline (1.0) represents Cr:Co in the pulse dose. Pigs were fed diets containing 500 g/kg rapeseed meal (RSM) or distillers dried grains with solubles (DDGS) as the only fiber source, with or without inclusion of β-glucan-extract (β-GLUC) or retrograded tapioca (RG). Error bars indicate SEM. An asterisk indicates differences from baseline (*P < 0.05).

Apparent ileal digestibility of non-glucosyl polysaccharides in the DDGS control diet was as expected [16], whereas that of RSM was less than observed previously [17] and found to be negative. Negative AID of NSP, particularly insoluble NSP fractions as cellulose and arabinoxylans, is reported more often and has been ascribed to the presence of endogenous and microbial material in ileal digesta and potential separation of marker and digesta [7, 40–45]. Considering the contribution of endogenous (i.e. mucus) and microbial material to ileal digesta (< 15%, w/w DM basis) and their non-glucosyl polysaccharides (mainly rhamnose, mannose, and galactose) contents [46–51], this would typically explain less than 2% of non-glucosyl polysaccharides found in ileal digesta. Provisional calculations indicate that correcting for endogenous and microbial material would increase ileal digestibility values by 2–3% units, but cannot fully explain the negative AID observed. More likely, collection of ileal digesta through the T-cannulas may have resulted in selective recovery of specific digesta fractions (see e.g. Köhler et al.[52]). Although not quantitatively analyzed, the ratio between chromium and cobalt (Cr:Co) in ileal digesta samples collected after pigs received a pulse-dose of Cr_2_O_3_ and Co-EDTA, provides some insight in the individual recoveries of solid and soluble digesta fractions. The Cr:Co supplied was 1.0, and deviations from this baseline indicate selective collection of specific fractions at that time-point. Averaged over 7 time-points in a period of 12 h from feeding, the Cr:Co of ileal digesta is expected to approximate to the baseline of 1.0, assuming recovery of the soluble and solid digesta fractions of the meal containing these markers is complete within 12 h [9]. Hence, the average Cr:Co of > 1 in ileal digesta for the RSM control diet (*P =* 0.036; Fig 1) indicates an overrepresentation of solid material in the digesta samples. Selective recovery of digesta fractions complicates digestibility measurements, particularly for fibrous components, due their low degradability and low solubility in ileal digesta. Even a slight overrepresentation of the high fibrous solid phase in ileal digesta could easily explain the negative ileal digestion coefficients for non-glucosyl polysaccharides. The discrepancy between the different diets may be related to physicochemical properties of the diets, such as solubility and water binding capacity of fiber fractions, which are influenced by the feed ingredients and additional fiber sources. Differences found for AID of non-glucosyl polysaccharides may therefore reflect differences in physicochemical properties among diets, rather than actual degradation of fiber fractions. Total tract degradation of residual glucosyl- and non-glucosyl polysaccharides from RSM were greater than those from DDGS, as expected [5, 15].

### Effect of added fibers on nutrient and fiber degradability

#### β-Glucans

Surprisingly, β-GLUC affected *in vitro* viscosity in the RSM diet much more than in the DDGS diet. We hypothesize that this observation can be explained by interactions between β-GLUC and polysaccharides in RSM. Approximately 25% of the added β-glucans were degraded before the end of the ileum, both in the RSM and the DDGS diet. This is lower than reported for barley β-glucans previously, when included at 3–5% in diets containing similar levels of NSP as the diets used in the present study [53–55]. It can be speculated that the abundance of recalcitrant polysaccharides in the diets of the present study led to a lower ileal degradation of β-glucans compared to diets with better degradable NSP. The ATTD of β-glucans was nearly complete, as expected [53–56].

Viscosity of ileal digesta measured after thawing at room temperature was not significantly affected by β-GLUC (*P* < 0.08). However, β-glucans may insolubilize during freezing (T. Vasanthan, personal communication) and freezing digesta samples may have masked effects of β-GLUC. Indeed, after heating of digesta samples, during which β-glucans are solubilized again, viscosity of β-GLUC diets was higher compared with control diets (18 and 233 mPa·s for RSM and DDGS diets respectively, *P* < 0.01).

The reduced AID of crude protein in β-GLUC diets can be partly contributed to the absence of caseinate in these diets. In the control diets, 10 (RSM diet) or 13% (DDGS diet) of the crude protein originated from caseinate. Even when correcting for the absence of caseinate in the β-GLUC diets, AID of crude protein is still 1 percentage unit lower than expected (calculated using AID of crude protein from RSM or DDGS and synthetic amino acids in control diets; AID for crude protein from β-GLUC assumed to be 70%), indicating that β-GLUC reduced AID of crude protein from RSM and DDGS.

The reduced AID of crude protein, crude fat, and starch indicate either increased endogenous or microbial matter in digesta [57] or that β-GLUC interferes with enzymatic digestion in the upper gastrointestinal tract. This can be explained by reduced enzyme accessibility [57, 58] or by a reduction in digesta retention time in the stomach and small intestine, as indicated by the reduced MRT of solid digesta (Table 3). Although, β-GLUC was expected to increase gastric retention time [19], small intestinal retention time may have been reduced, as only cumulative retention time in the stomach and small intestine was evaluated.

**Fig 2:**
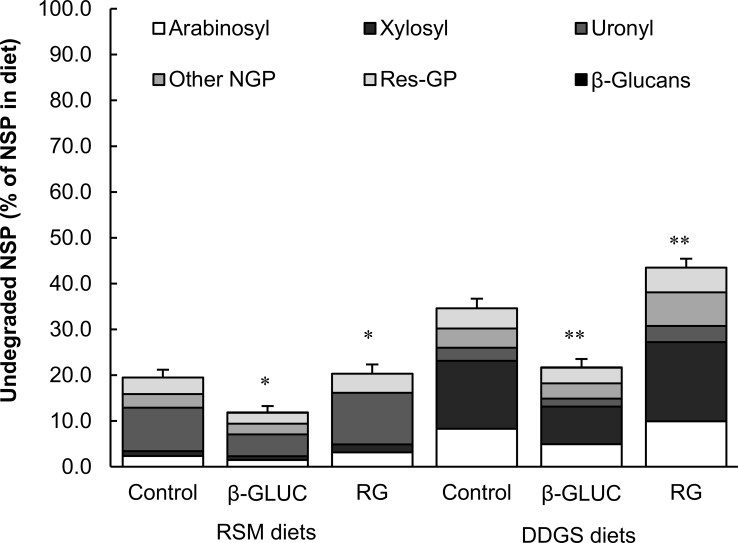
Undegraded Non-starch polysaccharides (NSP) in feces from growing pigs. Pigs were fed diets containing 500 g/kg rapeseed meal (RSM) or distillers dried grains with solubles (DDGS) as the only fiber source, with or without inclusion of β-glucan-extract (β-GLUC) or retrograded tapioca (RG). Error bars indicate SEM for total NSP. Asterisks indicate differences from control diet for total NSP *P* = 0.01; **P < 0.001). Non-starch polysaccharides are separated in β-glucans, residual glucosyl polysaccharides, arabinosyl, xylosyl, uronyl, and other non-glucosyl polysaccharides constituent sugars, where residual glucosyl polysaccharides are calculated as total glucosyl minus free glucose and glucosyl from starch and β-glucans, representing cellulose and other glucosyl-containing NSP.

Apparent total tract digestibility of non-glucosyl polysaccharides was higher in β-GLUC diets compared to the control diets, mainly because of increased degradation of xylosyl-polysaccharides (Table 2, Fig 2). Apart from β-glucans, the barley β-glucan-extract contained 23% (w/w) other NSP. These polysaccharides, that accounted for ~30% of the non-glucosyl polysaccharides in the RSM β-GLUC diet and for ~25% of the non-glucosyl polysaccharides in the DDGS β-GLUC diet, were presumably rather soluble, due to the nature of the extraction process. Assuming an ATTD of ~70% for these polysaccharides [53–55], it can be calculated that the degradation of non-glucosyl polysaccharides originating from DDGS itself was not affected by β-GLUC. The increased ATTD of non-glucosyl polysaccharides in the DDGS β-GLUC diet can thus be attributed to a better degradability of barley non-glucosyl polysaccharides compared with those from DDGS. This was expected based on the structural characteristics of the fiber-fractions [59]. Degradability of non-glucosyl polysaccharides from RSM, however, was still increased by β-GLUC, even after correction for the presence of barley non-glucosyl polysaccharides in the β-glucans extract. From the ATTD of constituent sugar moieties it follows that degradation of xylosyl-polysaccharides, likely originating mostly from xyloglucans [35], was affected (Table 2). Possibly, β-GLUC affected large intestinal retention time, allowing more time for fermentation and degradation of the rather recalcitrant xyloglucan-matrix. Alternatively, the presence of β-GLUC specifically stimulated colonization and activity of microbiota that possess activity towards RSM xyloglucans.

#### Resistant starch

*In vitro* viscosity of the diets and ileal digesta were not affected by RG addition. However, viscosity analyses were performed on supernatants of digesta such that insoluble polysaccharides, as resistant starch, were excluded. Ozone retrograded tapioca was reported to have a 2- to 3-fold higher viscosity than native corn starch [60]. Hence, it cannot be excluded that RG increased viscosity of diets and digesta, although not detected with the analytical procedures applied. The AID of starch of ~50% in RG diets indicates that indeed retrograded tapioca was not well degraded in the small intestine. Similar to β-GLUC, the addition of RG reduced retention time of solid digesta in the upper gastrointestinal tract, although this reduction was greater for the RSM than for the DDGS diet. Interestingly, RG reduced AID of crude protein in the DDGS diet but not in the RSM diet, whereas it increased AID of crude fat in the RSM diet only. As only retention time of solid digesta was affected by RG, differences between the nature and solubility of crude protein and fat in the RSM and DDGS diets and selective recovery of ileal digesta for the control RSM diet, may have contributed to these observations.

The greater amount of DM disappearance in the hindgut indicates that microbial activity and thus total amount of fermented matter was stimulated in RG diets, as expected [61]. However, RG reduced ATTD of non-glucosyl polysaccharides, both in RSM and the DDGS diet. The degradation of fibers from RSM and DDGS requires a complex system of cooperating and successive enzyme activities [35, 62, 63] and if RG reduced gastrointestinal retention time–as may be expected from the bulking properties of resistant starch [64, 65]–the reduced time available for fermentation may have limited the degradation of these recalcitrant fibers. Alternatively, the large amount of resistant starch that entered the hindgut undegraded was preferentially degraded by the microbiota over RSM and DDGS fibers. Preferential fermentation of resistant starch over other polysaccharides was also suggested for a wheat- and barley-based diet, although not quantitatively assessed [28].

**Fig 3:**
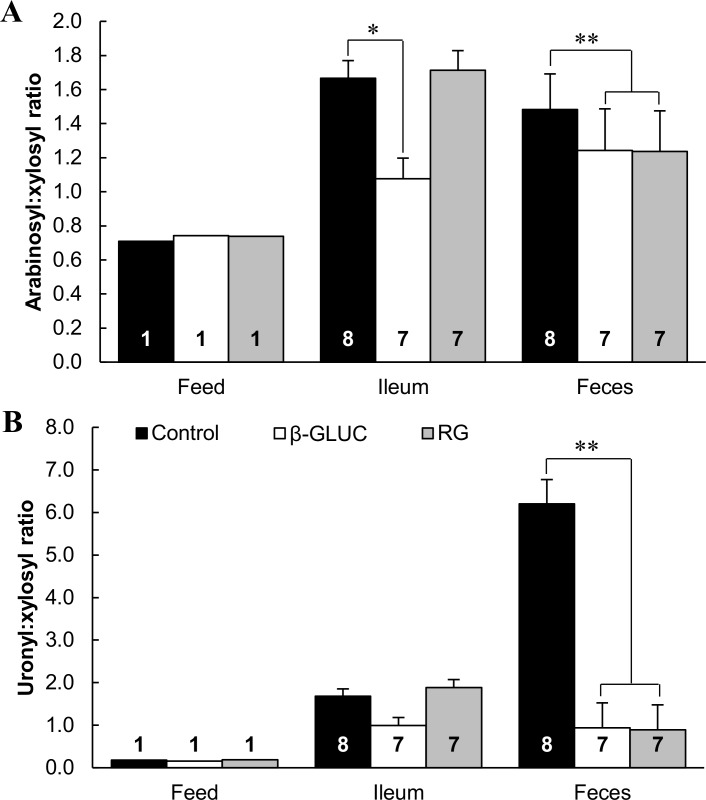
Arabinosyl:xylosyl and uronyl:xylosyl ratios in diets, ileal digesta, and feces from growing pigs. (A) Mean molar arabinosyl:xylosyl ratio. (B) Mean molar uronyl:xylosyl ratio. Pigs were fed diets containing 500 g/kg corn distillers dried grains with solubles as the only fiber source, with or without inclusion of β-glucan-extract (β-GLUC) or retrograded tapioca (RG). Data labels indicate number of replicate observations. Error bars indicate SEM. Asterisks indicate differences (*P = 0.05; **P < 0.01).

The ATTD of constituent sugar moieties indicates that arabinosyl-, xylosyl-, as well as uronyl- containing polysaccharides were affected by RG (Table 2). For DDGS, the ratio of arabinosyl and uronyl to xylosyl residues is indicative for the degree of substitution and thus related to the structure of the glucuronoarabinoxylans present in the feed or of the undegraded glucuronoarabinoxylans remaining in digesta and feces [16]. The lower arabinosyl:xylosyl as well as uronyl:xylosyl in feces from pigs fed the RG diets compared with pigs fed the control diets (Fig 3) indicates that especially degradation of the linear xylan backbone fragments was impaired in the RG diets. Degradation of the xylan backbone requires endoxylanase and β-xylosidase activities, which must be preceded by several debranching activities in order to gain access to the backbone structure [62, 63]. Possibly, reduced gastrointestinal retention time,–as expected from the bulking properties of resistant starch [64, 65]–, may have limited the time available for the cooperatively acting enzymes, resulting in less complete degradation of the complex xylan structures. Alternatively, it can be hypothesized that RG suppressed colonization and activity of specific microbiota that excrete xylan-degrading enzymes. Beside presumed higher microbial losses following the increased fermentation activity, the reduced apparent disappearance of crude protein in the hindgut in the RG diets compared with the control diets, suggests a reduced absorption of ammonium from the colon or increased influx of urea into the large intestine, shifting N excretion from urine to feces [66].

In conclusion, the presence of β-glucans and resistant starch altered digestion and fermentation of nutrients and fibers from other sources in the diet. The addition of 6% (w/w) β-GLUC to a RSM diet increased degradation of non-glucosyl polysaccharides from RSM (accounting for ~40% of the dietary fiber and 60% of the NSP fraction in RSM) by 6% units, whereas it had no effect on the degradation of non-glucosyl polysaccharides from DDGS. Addition of 40% (w/w) RG reduced degradation of non-glucosyl polysaccharides from RSM and DDGS diets by more than 10% units. Furthermore, AID of crude protein, fat, and starch were reduced when β-GLUC were added to the diet, corresponding to a reduced retention time of solid digesta in the upper gastrointestinal tract. Also addition of RG reduced retention time of solid digesta, the reduction being greater for the RSM than for the DDGS diet. Our results demonstrate that fermentation of fiber sources not only depends on fiber characteristics, but also on the presence of other fibers in the diet. Hence, interactions in the gastrointestinal tract among fibrous food or feed ingredients should be considered when evaluating their nutritive value.

## Supporting Information

S1 Table Analysed chemical compositions of canola meal, distillers dried grain with solubles from corn (DDGS), and fibre sources. (DOCX)

S2 Table Analysed and calculated sugar composition of experimental diets^*^ and starch-containing feed ingredients. (DOCX)
