# Supplementary material for: β-Glucans and Resistant Starch Alter the Fermentation of Recalcitrant Fibers in Growing Pigs
Source: PLoS One. 2016 Dec 2;11(12):e0167624. doi: 10.1371/journal.pone.0167624 (PMC5135129; doi:10.1371/journal.pone.0167624)
Supplement: S1 Table — (DOCX) [file pone.0167624.s001.docx]

**S1 Table. Analysed chemical compositions of canola meal, distillers dried grain with solubles from corn (DDGS), and fibre sources.**

| Item | Rapeseed meal | DDGS | β-GLUC | RG |
| --- | --- | --- | --- | --- |
| CP^*^, g/kg as-fed | 320 | 254 | 62 | n.a. |
| Total carbohydrates (CHO), g/kg as-fed | 257 | 356 | 725 | 898 |
| Starch | 3 | 45 | 238 | 825 |
| β-glucan | 0.5 | 4.3 | 267 | n.a. |
| Sugar composition of CHO, Mol%^†^ |  |  |  |  |
| Rhamnosyl | 2 | 0 | 0 | 0 |
| Arabinosyl | 20 | 19 | 7 | 0 |
| Xylosyl | 8 | 25 | 11 | 0 |
| Mannosyl | 3 | 5 | 2 | 0 |
| Galactosyl | 11 | 5 | 0 | 0 |
| Glucosyl | 39 | 42 | 78 | 99 |
| Uronyl | 16 | 5 | 1 | 1 |

DDGS, distillers dried grain with solubles; β-GLUC, β-glucan extract; RG, retrograded tapioca; n.a., not analysed; CHO, carbohydrates.

^*^ Crude protein content was calculated from the N content using a protein conversion factor of 5.3 for canola meal [1], 5.7 for β-GLUC [2], and 5.9 for DDGS [3].

^†^ Molar percentage; presented as anhydrous sugar moieties.

**References**

1. Mossé J. Nitrogen to protein conversion factor for ten cereals and six legumes or oilseeds. A reappraisal of its definition and determination. Variation according to species and to seed protein content. J Agric Food Chem. 1990;38:18-24.

2. Sosulski FW, Imafidon GI. Amino acid composition and nitrogen-to-protein conversion factors for animal and plant foods. J Agric Food Chem. 1990;38:1351-6.

3. Kim Y, Mosier NS, Hendrickson R, Ezeji T, Blaschek H, Dien B, et al. Composition of corn dry-grind ethanol by-products: DDGS, wet cake, and thin stillage. Biores Technol. 2008;99:5165-76.
