# Supplementary material for: β-Glucans and Resistant Starch Alter the Fermentation of Recalcitrant Fibers in Growing Pigs
Source: PLoS One. 2016 Dec 2;11(12):e0167624. doi: 10.1371/journal.pone.0167624 (PMC5135129; doi:10.1371/journal.pone.0167624)
Supplement: S2 Table — (DOCX) [file pone.0167624.s002.docx]

**S2 Table. Analysed and calculated sugar composition of experimental diets^*^ and starch-containing feed ingredients.**

|  | Rapeseed meal diets | | | DDGS diets | | | Feed ingredients | | |
| --- | --- | --- | --- | --- | --- | --- | --- | --- | --- |
| Item | Control | β-GLUC | RG | Control | β-GLUC | RG | β-GLUC | RG | Corn starch |
| Total glucosyl, % (w/w) | 48.1 | 47.4 | 50.8 | 53.5 | 54.9 | 56.5 | 58.8 | 88.4 | 86.7 |
| Free glucose | n.d.^†^ | 0.2 | n.d.^†^ | 0.3 | 0.6 | 0.5 | 1.17 | n.d.^†^ | 0.02 |
| Starch | 37.7 | 28.3 | 41.1 | 42.0 | 31.5 | 43.8 | 23.8 | 82.5 | 71.2 |
| β-glucan | 0.1 | 5.3 | 0.1 | 0.3 | 5.8 | 0.3 | 26.7 | n.a. | n.a. |
| Residual glucosyl polysaccharides^‡^ | 5.5 | 8.9 | 4.6 | 5.6 | 11.6 | 6.3 | - | - | - |
| Calculated NSP content^§^ | 12.7 | 22.4 | 12.7 | 15.3 | 25.0 | 15.3 | - | - | - |
| Total sugar in NSP extract^‖^, % (w/w) | 9.6 | 18.2 | 9.4 | 14.1 | 20.5 | 13.5 | n.a. | n.a. | n.a. |
| Glucosyl | 3.1 | 9.0 | 3.2 | 4.4 | 9.8 | 4.3 | n.a. | n.a. | n.a. |
| of which starch | 0.04 | 0.29 | 0.10 | 0.13 | 0.17 | 0.12 | n.a. | n.a. | n.a. |
| NSP glucosyl not analysed^¶^ | 2.0 | 3.4 | 2.0 | 1.2 | 2.9 | 1.3 | - | - | - |
| Non-glucosyl polysaccharides | 6.4 | 9.2 | 6.2 | 9.7 | 10.7 | 9.2 | n.a. | n.a. | n.a. |
| Not analysed^**^ | 1.1 | 1.1 | 1.4 | 0.1 | 1.8 | 0.6 | n.a. | n.a. | n.a. |

DDGS, distillers dried grain with solubles; β-GLUC, β-glucan extract; RG, retrograded tapioca; n.d., not detected; n.a., not analysed; NSP, non-starch polysaccharides.

^*^ Diets contained either 500 g/kg canola meal or corn distillers dried grain with solubles (DDGS). Corn starch (control diet) was either replaced with β-glucan extract (β-GLUC; ~60 g/kg, as-fed basis) or retrograded tapioca (~400 g/kg, as-fed basis).

^†^ Not detected, with detection limit at 0.2 g/100g.

^‡^ Calculated as total glucosyl minus free glucose and glucosyl from starch and β-glucan.

^§^ Expected NSP content of diets calculated based on NSP contents of individual feed ingredients.
